# Hemidiaphragmatic Paralysis Post Stroke Leading to Hypercapneic Respiratory Failure

**DOI:** 10.7759/cureus.13141

**Published:** 2021-02-04

**Authors:** Antony J Arumairaj, Sanket Agarwal, Rachana Borkar, Hansang Park, Imnett Habtes

**Affiliations:** 1 Internal Medicine, Metropolitan Hospital Centre, New York, USA; 2 Internal Medicine, Metropolitan Hospital Center, New York, USA; 3 Internal Medicine, New York Medical College, Metropolitan Hospital Center, New York, USA; 4 Pulmonary and Critical Care Medicine, Metropolitan Hospital Center, New York, USA

**Keywords:** diaphragmatic paralysis, non invasive ventilation, hypercapneic respiratory failure, stroke, pneumonia

## Abstract

Unilateral diaphragmatic paralysis is a rare complication after stroke. We report a case of right-sided hemidiaphragmatic paralysis after stroke in a 51-year-old man who presented with shortness of breath and orthopnea. Chest X-ray (CXR) revealed an elevated right-sided hemidiaphragm. The weakened diaphragmatic contraction from paralyzed right hemidiaphragm resulted in persistent atelectasis of the right lung base and inadequate alveolar ventilation leading to the development of right basal pneumonia with hypercapneic respiratory failure. However, the patient had a remarkable improvement with the appropriate institution of non-invasive ventilation and medical management with intravenous antibiotics.

## Introduction

The diaphragm plays a vital role in respiration. Contraction of the diaphragm creates a negative intrathoracic pressure leading to inspiration. At the end of inspiration, the diaphragm relaxes and increases the intrathoracic pressure leading to expiration [1]. Diaphragmatic paralysis is one of the critical causes of loss of control of diaphragmatic function. Diaphragmatic paralysis could be unilateral or bilateral. The causes for hemidiaphragmatic paralysis are phrenic nerve compression from lung cancer, surgical trauma to the phrenic nerve from cardiothoracic and cervical spine procedures, birth trauma, and neurologic disorders like amyotrophic lateral sclerosis, multiple sclerosis, and muscular dystrophy [2]. Depending on the cause and severity of the paralysis and whether the weakness is either unilateral or bilateral, the symptoms of diaphragmatic paralysis vary from being entirely asymptomatic to disabling dyspnea [3]. Hemidiaphragmatic paralysis after stroke is an uncommon complication that can be missed until the very end when the patient develops marked respiratory failure and presents to the emergency department. We report a rare case of hypercapneic respiratory failure secondary to the right hemidiaphragm paralysis after stroke.

## Case presentation

**Figure 1:**
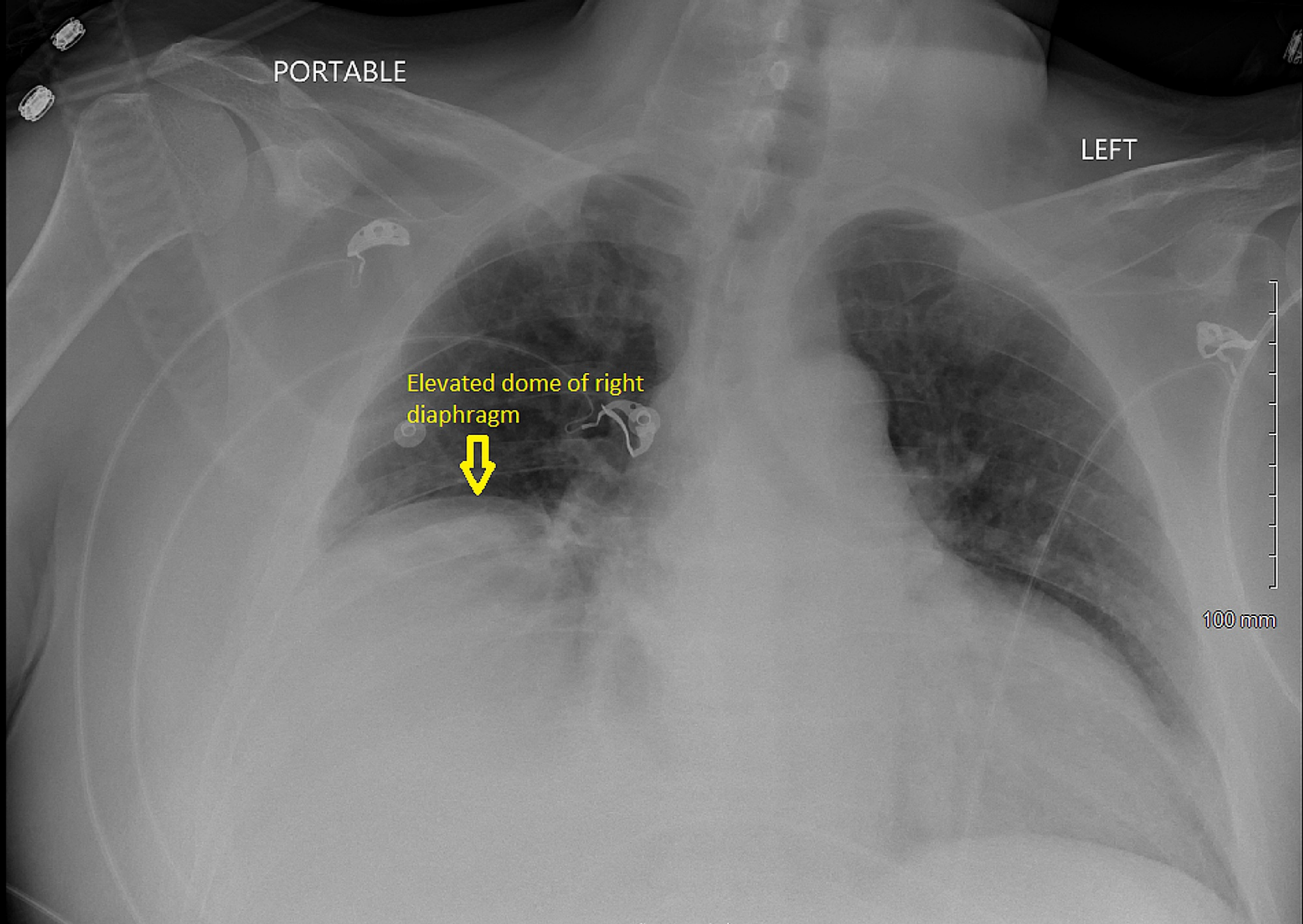
Anteroposterior chest X-ray depicting the elevation of right-sided hemidiaphragm post stroke.

**Figure 2:**
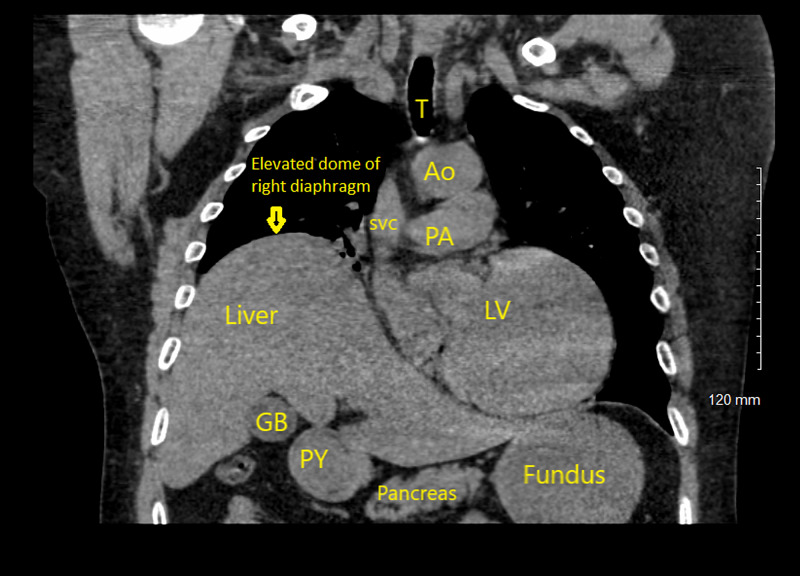
Coronal section of non-contrast CT Chest depicting the elevated dome of right hemidiaphragm with adjacent structures of gall bladder (GB) , pylorus of stomach (PY), left ventricle (LV), trachea (T), aorta (Ao), pulmonary artery (PA) and superior vena cava (SVC).

**Figure 3:**
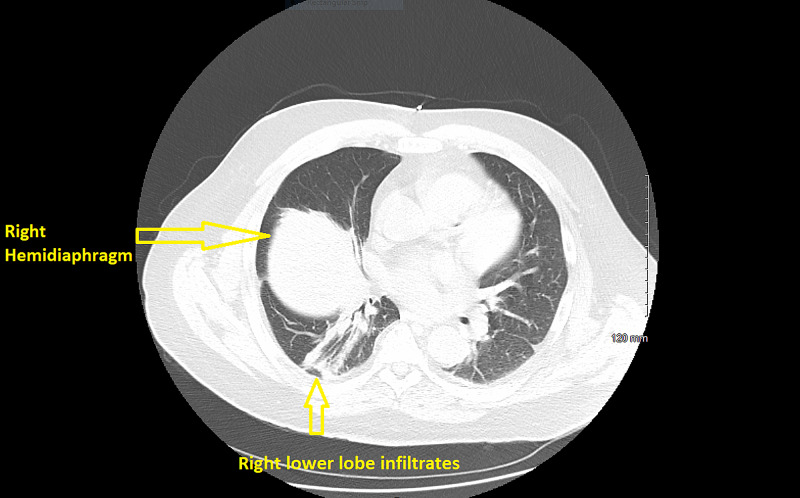
Axial section of the non-contrast CT chest demonstrating the right lower lobe infiltrates and elevated right hemidiaphragm.

**Figure 4:**
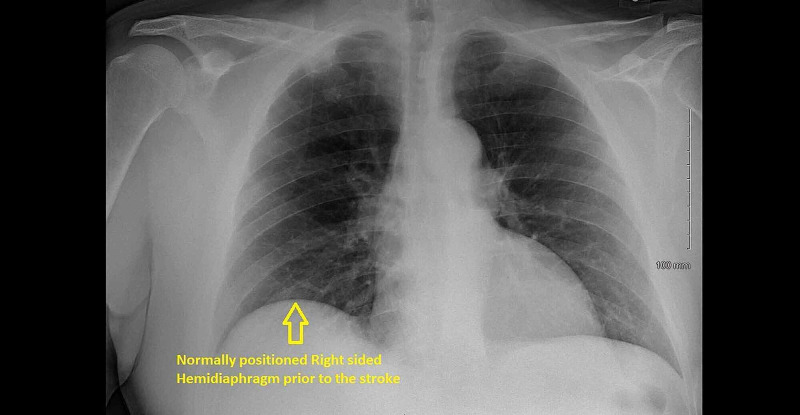
Anteroposterior chest X-ray depicting a normally positioned right-sided hemidiaphragm prior to the stroke.

**Figure 5:**
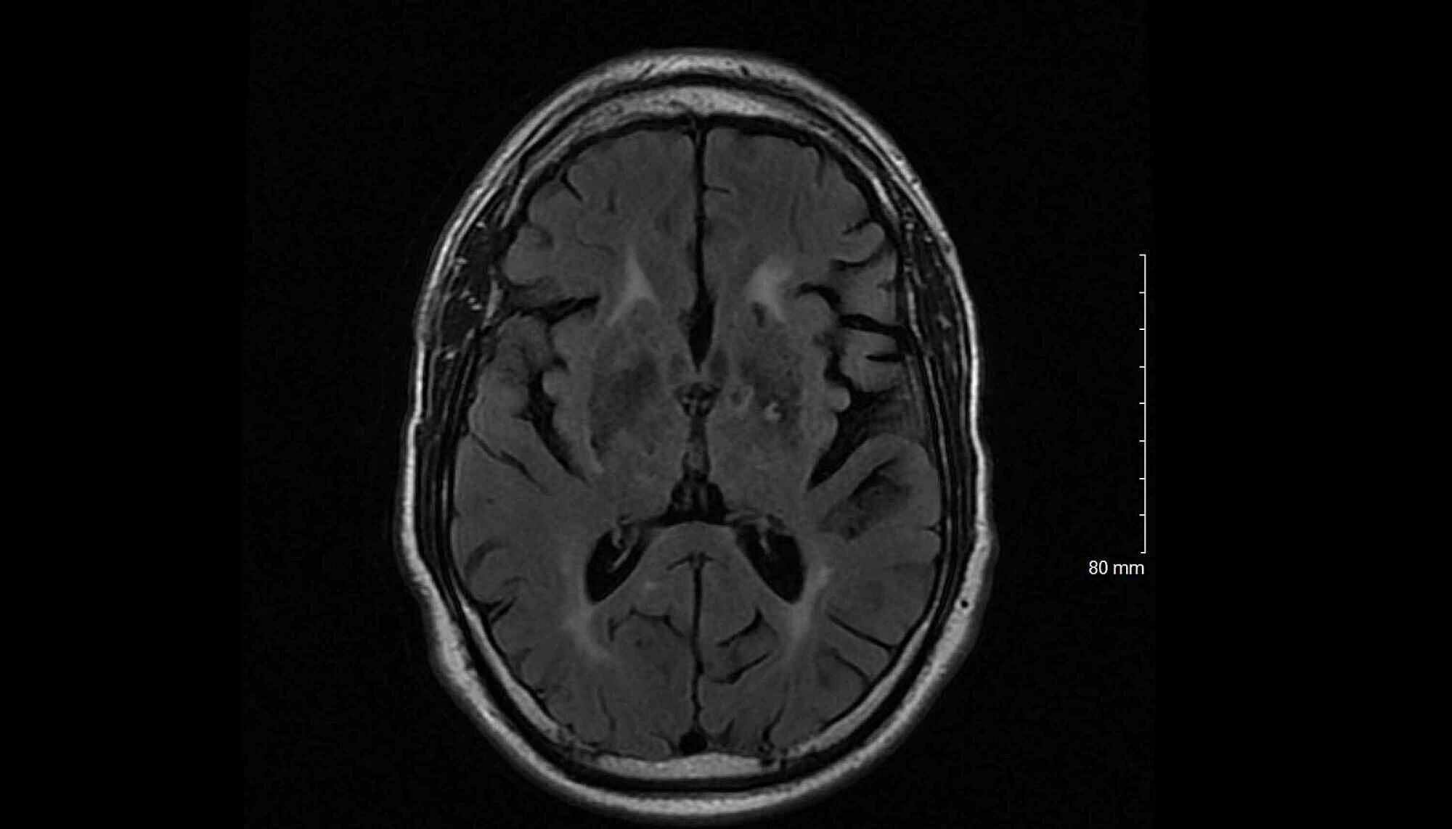
Non-contrast MRI brain shows hypointensity in the posterior limb of the left internal capsule consistent with an infarct and bilateral white matter hyperintensities consistent with microvascular changes in axial T2 FLAIR imaging. FLAIR: fluid-attenuated inversion recovery.

A 51-year-old man with a past medical history of hypertension, hyperlipidemia, chronic kidney disease (CKD) stage three, and multiple lacunar strokes with severe cognitive impairment was brought in to the emergency department (ED) with one week duration of gradually worsening of shortness of breath (SOB), dizziness, orthopnea requiring three pillows to sleep, and a reduced exercise tolerance of less than one block. On arrival, the patient was found to be in marked respiratory distress with a respiratory rate of 32 per minute, oxygen saturation of 82 percent on room air, heart rate of 110 beats per minute, and blood pressure of 161/101 millimeters of mercury (mmHg). Chest examination revealed reduced air entry in the right lung base. Neurological examination revealed severe cognitive impairment, dysarthria, and residual deficits of left-sided facial palsy and right arm weakness from the previous infarcts. Initial arterial blood gas (ABG) analysis showed a pH of 7.26, partial pressure of oxygen (PaO2) of 78 mmHg, and partial pressure of carbon dioxide (PaCO2) of 68 mmHg consistent with respiratory acidosis from hypercapneic respiratory failure. Laboratory results showed an elevation of white cell count to 14 x 10^3^/microliter (mcl). A chest X-ray (CXR) revealed elevation of the right-sided hemidiaphragm (Figure 1). Non-contrast computed tomography (CT) of the chest confirmed the elevated right-sided hemidiaphragm (Figure 2) with the right basal consolidation suggestive of pneumonia (Figure 3). However, the CXRs done in the previous admission before the stroke showed a normally placed right and left hemidiaphragm (Figure 4). Non-contrast CT of the cervical spine was normal and showed no evidence of cervical spine fracture, spondylosis, or disc herniation to suggest cervical nerve root compression. The patient had an admission for a stroke two years ago. Magnetic resonance imaging (MRI) of the brain done at that time showed bilateral basal ganglia, thalamic, external capsule, cerebellar, and left internal capsule lacunar infarcts (Figure 5). 

The diagnosis was hypercapneic respiratory failure secondary to a right-sided hemidiaphragmatic paralysis with right basal pneumonia. The patient was started on bilevel positive airway pressure (BiPAP) ventilation and was admitted to the medical intensive care unit. BiPAP settings were inspired positive airway pressure (IPAP) of 12 centimeters (cm) of water (H2O), expired positive airway pressure (EPAP) of 6 cm of H2O, and a fraction of inspired oxygen (FiO2) of 40%. The patient was started on broad-spectrum intravenous (IV) antibiotics with a renal adjusted dose of cefepime and azithromycin. The patient was continued on his regular medications of aspirin, toprol, lisinopril, atorvastatin, and amlodipine. The repeat ABG analysis showed improving respiratory acidosis with a pH of 7.33, PaO2 of 162 mmHg, and PaCO2 of 48 mmHg. The patient continued to show symptomatic improvement, and dyspnea resolved with BiPAP ventilation and completion of the course of IV antibiotics. Improvement in respiratory acidosis was monitored with regular ABG analysis. Nocturnal BiPAP ventilation was continued throughout the hospital stay. The patient was discharged after ten days of treatment.

The patient was advised to continue long term night time BiPAP ventilation after discharge to prevent hypoventilation and recurrent hypercapneic respiratory failure. The patient was discharged to his home with long term BiPAP ventilation. The patient was followed in the pulmonary clinic and was compliant with the nocturnal BiPAP ventilation, and has not had any further admissions again with respiratory failure.

There were no recent cardiothoracic and cervical spine surgeries, nor was there any evidence of lung malignancy in the CT scans to suggest the phrenic nerve injury and compression as a cause of right-sided hemidiaphragmatic paralysis. The possibility of cervical nerve root compression was also less likely from the normal CT cervical spine. There was no evidence of lower motor neuron (LMN) signs to suggest amyotrophic lateral sclerosis, nor was there any evidence of plaques and demyelination in the MRI Brain to suggest multiple sclerosis. There was no history of progressive muscle weakness to suggest muscular dystrophy. Lacunar stroke with infarct involving the posterior limb of the internal capsule emerged as the most likely cause of right hemidiaphragmatic paralysis. The CXRs demonstrating the normally placed right hemidiaphragm before the stroke and the elevated right hemidiaphragm after the stroke further reinforced that the stroke was the likely cause of the right hemidiaphragmatic paralysis.

## Discussion

Stroke with a lacunar infarct in the internal capsule, as in our patient, is an unusual cause of hemidiaphragm paralysis [4]. Ascending and descending fiber tracts transit within the internal capsule to connect the cerebral hemispheres with subcortical structures, the brainstem, and the spinal cord. The internal capsule is prone to cerebrovascular accidents because the perforating arteries that supply the region are predisposed to occlusion due to their small diameter. Ischemic strokes secondary to blockage of the perforating arteries are known as lacunar strokes [5,6]. The posterior limb of the internal capsule contains the critical corticospinal tract that provides direct inputs onto phrenic motor neurons for contralateral diaphragmatic contraction and volitional control of breathing. A lacunar stroke in the posterior limb of the internal capsule can affect the corticospinal tract, which results in contralateral hemidiaphragmatic paralysis [4,7]. Assessment of diaphragmatic movements and corticodiaphragmatic pathway in ischemic stroke patients has shown abolishment or retardation of diaphragm contraction [8-10]. This case demonstrates the critical impact of a lacunar stroke in the posterior limb of the internal capsule resulting in contralateral hemidiaphragmatic paralysis and hypercapneic respiratory failure.

The diaphragm contributes up to 60%-70% of the total ventilation at rest, in both sitting and supine positions. Hemidiaphragmatic paralysis results in a vital capacity decrement of 10%-30%, with the most substantial decrements seen in the supine position [11,12]. The reduction in vital capacity explains the incidence of orthopnea in patients after diaphragmatic paralysis.

The right-sided hemidiaphragmatic paralysis resulted in a weakened right diaphragmatic contraction and an elevated right-sided hemidiaphragm, which impaired the excursion of the hemidiaphragm with each breath resulting in alveolar hypoventilation and impaired gas exchange leading to hypercapneic respiratory failure [13]. The diaphragmatic paralysis and elevated right hemidiaphragm also contributed to the development of right basal pneumonia by causing persistent atelectasis of the right lung base. The decreased ability to cough and decreased mucociliary transport secondary to the impairment of contraction of the right hemidiaphragm led to the accumulation of mucus in the right lung base, which served as a nidus leading to the subsequent development of right basal pneumonia [14-16].

Clinical examination is usually not significant in diaphragmatic paralysis except in severe bilateral diaphragmatic paralysis wherein paradoxical respiratory movements can be seen. Around 90% of hemidiaphragmatic paralysis is diagnosed based on an elevated hemidiaphragm on routine CXRs. CT of the chest should be done in all patients with hemidiaphragmatic paralysis to rule out any thoracic pathology causing the phrenic nerve compression [17,18].

The treatment of hemidiaphragmatic paralysis depends on the cause of the paralysis and the degree of impairment in respiratory functions [18]. Asymptomatic patients do not need any interventions but will have to be closely followed up for early signs and symptoms of respiratory failure [19]. Patients who develop respiratory failure will need long term non-invasive ventilatory assistance with BiPAP. Surgical intervention with diaphragmatic plication is indicated in patients who fail to improve after conservative treatment with BiPAP [11,19]. In this procedure, the flaccid hemidiaphragm is made taut by oversewing the membranous central tendon and the muscular peripheral part of the diaphragm. This procedure reduces the normal contralateral hemidiaphragm's workload and improves ventilation/perfusion of the affected lung base. Diaphragmatic plication has been shown to decrease dyspnea and improve functional capacity [20].

In patients with bilateral diaphragmatic paralysis, the initial treatment is non-invasive ventilatory assistance with BiPAP. When non-invasive ventilation fails, tracheostomy with mechanical ventilation will be required. There is no role for diaphragmatic plication in bilateral diaphragmatic paralysis. However, patients with bilateral diaphragmatic paralysis with persistent respiratory failure and intact phrenic nerve may benefit from phrenic nerve pacing [18].

## Conclusions

The incidence of pneumonia and hypercapneic respiratory failure secondary to hemidiaphragmatic paralysis in patients after stroke can significantly increase the mortality and morbidity of affected patients, especially those with poor cardiopulmonary reserve. Hence a high index of clinical suspicion is needed to identify early signs and symptoms of hemidiaphragmatic paralysis after stroke. The timely initiation of broad spectrum antibiotics and BiPAP ventilation in our patient proved to be crucial in the resolution of the right basal pneumonia and in reversing the effects of hypercapneic respiratory failure, thereby preventing intubation and potential complications of prolonged invasive ventilation in patients after stroke.
